# Discovery of a small molecule that inhibits Bcl-3-mediated cyclin D1 expression in melanoma cells

**DOI:** 10.1186/s12885-023-11663-y

**Published:** 2024-01-18

**Authors:** Karunakar Saamarthy, Kristofer Ahlqvist, Renée Daams, Navisraj Balagunaseelan, Agnes Rinaldo-Matthis, Julhash U. Kazi, Wondossen Sime, Ramin Massoumi

**Affiliations:** 1 Department of Laboratory Medicine, Translational Cancer Research, Division of Molecular Tumor Pathology, Lund University, Medicon Village, 22383 Lund, Sweden; 2 Department of Medical Biochemistry and Biophysics, Division of Chemistry II, Karolinska Institutet, Stockholm, Sweden

**Keywords:** Melanoma, Bcl-3, Cyclin D1, Proliferation

## Abstract

Molecular targeted therapy using a drug that suppresses the growth and spread of cancer cells via inhibition of a specific protein is a foundation of precision medicine and treatment. High expression of the proto-oncogene Bcl-3 promotes the proliferation and metastasis of cancer cells originating from tissues such as the colon, prostate, breast, and skin. The development of novel drugs targeting Bcl-3 alone or in combination with other therapies can cure these patients or prolong their survival. As a proof of concept, in the present study, we focused on metastatic melanoma as a model system. High-throughput screening and in vitro experiments identified BCL3ANT as a lead molecule that could interfere with Bcl-3-mediated cyclin D1 expression and cell proliferation and migration in melanoma. In experimental animal models of melanoma, it was demonstrated that the use of a Bcl-3 inhibitor can influence the survival of melanoma cells. Since there are no other inhibitors against Bcl-3 in the clinical pipeline for cancer treatment, this presents a unique opportunity to develop a highly specific drug against malignant melanoma to meet an urgent clinical need.

**Supplementary Information:**

The online version contains supplementary material available at 10.1186/s12885-023-11663-y.

## Introduction

Melanoma is an aggressive type of skin cancer originating from neural crest-derived melanin-producing cells in the basal layer of the epidermis [1]. Malignant melanoma is the deadliest type of skin cancer, and the median survival time for stage IV melanoma patients ranges from 8–18 months postdiagnosis [2]. However, due to recent advancements in targeted therapies and immunotherapies, the overall survival time of patients with melanoma is increasing [3, 4]. Melanomas are very heterogeneous diseases harboring several somatic mutations, including mutations found in BRAF, NRAS, NF1, KIT, and PTEN, among others, which can predict the clinical efficacy of targeted therapies [5, 6]. Mutations in cell cycle genes, such as CDKN2A, RB1, MDM2, and TP53, often result in faster cell cycle transitions, leading to increased cell proliferation [5, 7, 8]. Uncontrolled cell proliferation is a key hallmark of cancer and is achieved through several mechanisms, including elevated levels of cyclin D1 expression. Cyclin D1 regulates the G1-S transition of the cell cycle through phosphorylation of the retinoblastoma (Rb) protein together with CDk4/6 and is often overexpressed or amplified in many types of cancer [9].

One mechanism through which cyclin D1 expression is regulated is by direct binding of the nuclear factor kappa B (NFκB) transcription factor family to this promoter, thereby controlling gene transcription [10]. The NFκB signaling pathway is involved in many cellular processes, including immune/inflammatory responses, cell proliferation, cell growth, cell survival, and migration [11]. The NFκB family of transcription factors consists of five proteins: RelA (p65), RelB, c-Rel, p105/p50, and p100/p52, which interact with one another by forming homodimers or heterodimers. These proteins share a common domain called the Rel homology domain (RHD), which is necessary for dimerization, interaction with inhibitor of NFκB (IκB) proteins, nuclear translocation, and DNA binding [12]. NFκB signaling is tightly regulated through IκB proteins and IκB kinase (IKK) proteins that phosphorylate the IκB proteins, causing their degradation. Unlike canonical IκBs that sequester NFκB proteins in the cytoplasm, atypical IκB B-cell lymphoma 3 (Bcl-3) has both has inhibitory and activating functions [12, 13].

Bcl-3 was originally identified as a gene involved in the recurring chromosomal translocation t(14;19), which is found in patients with chronic lymphocytic leukemia [14]. Since the discovery that Bcl-3 is an oncogene involved in leukemia, other studies have demonstrated the oncogenic function of Bcl-3 in solid tumors originating from breast [15–17], liver [18], prostate [19], colon [20–23], cylindromatosis [24], basal cell carcinoma [25], squamous cell carcinoma [26], and nasopharyngeal carcinoma [27]. In transgenic animals, sustained Bcl-3 nuclear translocation leads to the development of skin papilloma [24] or squamous cell carcinoma [26] by augmentation of p50/p52-related NF-κB binding in mouse keratinocytes. It has also been shown that p53 decreases the expression of Bcl-3, which changes the ratio of p52/Bcl-3 to p52/HDAC complexes on the cyclin D1 promoter, thus inhibiting cyclin D1 expression [28]. Furthermore, in melanoma Bcl-3 promotes the migration and invasion of these cells through upregulation of Snail and Slug proteins, which play a critical role in the epithelial to mesenchymal (EMT) process [29]. Previously, it has been shown that Bcl-3 can directly activate the cyclin D1 promoter by binding to an NFκB binding site together with p52 homodimers. The recruitment of Bcl-3 to the cyclin D1 promoter resulted in a shorter G1 cell cycle phase and hyperphosphorylation of Rb protein [30].

Although Bcl-3 is a well-known proto-oncogene and is dysregulated in many types of cancer, thus far, no therapeutic agents have been developed targeting Bcl-3 to regulate its effects. In the present study, we aimed to identify a Bcl-3 inhibitor that could interfere with Bcl-3-mediated cyclin D1 promoter activity. We identified a small molecule that inhibited cyclin D1 expression and proliferation in melanoma cells and suppressed tumor growth in animals.

## Materials and methods

### Cell culture and reagents

Melanoma cancer cell line Mel Juso was cultured in DMEM that was supplemented with 1 g/L Glucose (Corning), 10% FBS (Biosera), and 100 U/ml penicillin/streptomycin (Corning). Melanoma cell lines: Mel Ju, A7, HMCB, WM239A, WM852, and A2058 were cultured in RPMI-1640 supplemented with 10% FBS and 100 U/ml penicillin/streptomycin (Corning). SK-MEL-3 and HT144 cells were cultured in McCoy’s 5A medium supplemented with 10% FBS (Biosera), 100 U/ml penicillin/streptomycin (Corning). Mel Juso, Mel Ju, Mel Ho, and SK-MEL-3 cells were kindly provided by Prof. Anja Bosserhoff (Friedrich-Alexander University Erlangen-Nuremberg, Germany). A7, HMCB, WM239A, WM852, HT144, and A2058 HT144 cells were kindly provided by Prof. Göran Jönsson (Lund University, Sweden). All cell lines were cultured at 37 °C in a humidified atmosphere containing 5% CO_2_. Cells were trypsinized with 0.25% trypsin (Corning, Cat No. 25–053-CI). Cell Line authentication has been performed for all the cell lines presented in this study. Immortalized keratinocytes were cultured and transfected as described previously [24]. BCL3ANT was dissolved in DMSO at different concentrations and stored at -20 °C until use.

### Plasmids

p-Bcl-3-FLAG was kindly provided by Dr. Roland Schmid, Munich Technical University, Munich, Germany. Cyclin D1 promoter and NFκB mutant cyclin D1 promoter were kindly gifted by Dr. Richard G. Pestell (Pennsylvania Cancer and Regenerative Medicine Research Center, Baruch S. Blumberg Institute, Wynnewood, PA, USA). The NFκB site in the cyclin D1 promoter at 239 to 230 base pairs (bp) was mutated by a polymerase chain reaction from 5’-GGG GAG TTT T-3’ to 5’-GCC CAG TTT T-3’.

### Production and purification of GST-Bcl3-ARD

#### Protein production

4 L of Luria Broth (Difco) supplemented with 100 μg/ml ampicillin was inoculated to OD600 = 0.1 with an overnight culture of E. coli TUNER / pGEX-2 T-PreScission-Bcl3-ARD (plasmid made by LP3). The culture was grown in 5 L Ehrlenmeyer flasks with indentations (1 L / flask) at 30 °C, 120 rpm. At OD600 = 0.8, IPTG was added to a final concentration of 1 mM. Three hours after induction, the cells were harvested in a JLA 8.1000 rotor, 8000xg, 4 °C, 15 min, and the pellets were stored at -80 °C.

#### Purification of GST-Bcl3-ARD

Cell pellets from 4 L E. coli TUNER / pGEX-2 T-PreScission-Bcl3-ARD culture was resuspended in 100 ml PBS, pH 7.3, supplemented with 4 tablets Complete Protease Inhibitor, EDTA-free (Roche). The cell suspension was passed twice through a French Pressure cell at 18 000 psi. The lysate was ultracentrifuged in a Ti 50.2 rotor, 45 000 rpm, 60 min, 4 °C, and the supernatant (soluble fraction) was passed through a 0.45 μm filter. The soluble fraction was used for affinity chromatography. A Protino GST/4B 5 ml column was connected to an ÄKTA Avant system. The column was run at room temperature, while fractions were collected at 6 °C. The column was equilibrated with 5 column volumes (CV) ddH2O at a flow rate of 1 ml/min followed by 2 CV PBS, pH 7.3 at 2 ml/min. The sample was then applied at a flow rate of 0.5 ml/min using the air sensor. The column was washed with PBS, pH 7.3 until a stable UV signal was obtained (flow rate 5 ml/min), and bound protein was eluted with 10 CV 50 mM Tris–HCl, 20 mM reduced glutathione, pH 8.0 at a flow rate of 1 ml/min. The flow through and wash fractions were saved, and during elution, 5 ml fractions were collected in 15 ml tubes already containing 5 ml buffer (50 mM Tris–HCl, 150 mM NaCl, pH 8.0) to avoid precipitation of the protein. Fractions were pooled and dialyzed to 50 mM Tris–HCl, 150 mM NaCl, pH 8.0 at 4 °C overnight to get rid of the glutathione. Samples from the purification of GST-Bcl3-ARD were analyzed on a Mini-Protean TGX Precast Any kD SDS-PAGE gel (Bio-Rad) stained with Bio-Safe Coomassie (Bio-Rad).

#### Protein concentration and delivery

The concentration of GST-Bcl3-ARD in the dialyzed pool was measured in a NanoDrop Spectrophotometer. The final concentration of GST-Bcl3-ARD were usually between 10–15 mg/ml in a total volume of 30 ml. When the protein was precipitated at high concentrations (15 ml) it was diluted to 2.2 mg/ml with 50 mM Tris–HCl. 150 mM NaCl, pH 8.0.

#### Purification of cleaved Bcl3-ARD

100 mg GST-Bcl3-ARD was dialyzed to 100 mM NH4OAc pH 6.0, 150 mM NaCl. PreScission was added to an enzyme:substrate ratio of 1:200. Also, DTT was added to a final concentration of 1 mM before the reaction was incubated at 4 °C overnight.

#### Affinity chromatography to remove GST-tag and PreScission

After PreScission digestion, the sample was loaded onto a Protino GST/4B 5 ml column, connected to an ÄKTA Avant system (GE Healthcare). The column was washed with 5 CV ddH2O at 1 ml/min, and was then equilibrated with 5 CV 100 mM NH4OAc pH 6.0, 150 mM NaCl at 2 ml/min. The sample was applied using a flow rate of 0.5 ml/min, and the column was washed with 2 CV 100 mM NH4OAc pH 6.0, 150 mM NaCl (flow rate 2 ml/min). The bound GST-tag and PreScission were eluted with 5 CV of 50 mM Tris–HCl pH 8.0, 150 mM NaCl, 20 mM glutathione using a flow rate of 2 ml/min. The flow through, wash and elution fractions were collected. The chromatography run was performed at room temperature while the fractions were collected at 6 °C. Samples from the affinity chromatography of cleaved Bcl3-ARD batch 3 were analyzed on a Criterion TGX Precast Any kD SDS-PAGE gel (Bio-Rad) stained with Bio-Safe Coomassie (Bio-Rad).

#### Size exclusion chromatography

The flow through and wash fraction from the affinity chromatography were pooled (22 ml) and concentrated to 10 ml using a 15 ml Amicon centrifugal cellulose membrane filter (10 kDa MWCO). The concentrated sample was passed through a 0.2 μm filter before loading it using a 10 ml superloop onto a 320 ml HiLoad 26/600 Superdex 75 pg gel filtration column (GE Healthcare) connected to an ÄKTA Purifier system. The column was run at 4 °C, with a flow rate of 2.6 ml/min. 100 mM NH4OAc pH 6.0, 150 mM NaCl was used as running buffer and 5 ml fractions were collected. Samples from the collected fractions were analyzed on a Criterion TGX Precast Any kD SDS-PAGE gel (Bio-Rad) stained with Bio-Safe Coomassie (Bio-Rad). The concentration Bcl3-ARD in the three different pools from the size exclusion chromatography was measured in a NanoDrop spectrophotometer.

### Transfections

Transient transfections were performed in 96-well plates using PolyFect reagent (Qiagen) according to the manufacturer’s instructions. Cells were transfected with a total of 470 ng DNA consisting of: Renilla (20 ng), cyclin D1 promoter (400 ng) or NFκB mutant cyclin D1 promoter (400 ng), together with empty vector (50 ng, Clonetech), or p-Bcl-3-FLAG (50 ng). Cells were transfected for 24 h, after which they were treated with or without drugs for 24 h. Following incubation with or without drugs, cells were washed twice with PBS and analyzed using the Dual-Luciferase Reporter Assay System kit, from now on referred to as: Dual Reporter Assay (Promega) according to the manufacturer’s instructions. Luciferase activity was measured on the Synergy 2 HT Multi-Mode Microplate Reader (Biotek). All experiments were performed three times in triplicates and an average DLR for the control was set to 1. siRNAs (control and Bcl-3) were purchased from Santa Cruz and used according to the manufacturer's instructions. siRNA transfections were made with HiPerFect transfection reagent (QIAGEN).

### High-throughput screening

Mel Juso cells were seeded on 96-well plates at a density of 2 × 10^4^ cells per well (day 0). After cells were attached, transfections were conducted for 24 h using the following plasmids: p-Bcl-3-FLAG, together with reporter plasmid cyclin D1 Luciferase and Renilla-Luc (Day 1). Compounds (Diversity Set, 1368 compounds; National Cancer Institute, Bethesda, MD) were added at a final concentration of 10 μM in DMSO (day 2). Cells were incubated for 24 h, lysed, and analyzed using the Dual Reporter assay (Promega). All experiments were performed three times in triplicates. To assay for transfection efficiency, transfections were performed with a constant amount of Renilla expression plasmid. Luciferase and Renilla activities were assayed 24 h post-transfection according to the Dual Reporter assay manufacturer’s instructions.

### Thermal Stability Assay (TSA)

Real-time PCR device CFX connect (BioRad) was used to monitor protein unfolding through the increase in fluorescence of fluorophore SYPRO Orange (Invitrogen). Protein samples (GST-Bcl-3-ARD, 5 μg in 25 μl) were diluted in buffer containing 50 mm Tris–HCl pH 8.0, 150 mM NaCl, and incubated on 96-well microplates in the RT-PCR device in the presence of SYPRO Orange and ligand (final concentration of 10 μM). The ligand stock of 10 mM was dissolved in 100% DMSO. Optical foil was used to cover the microplates. Samples were heated at 1 °C per minute, from 20 °C to 80 °C. Fluorescence intensity was measured every 1 °C and plotted as a function of temperature by using the software package within the BioRad instrument. Each point represents the negative derivative or the negative slope at each temperature. The Tm (melting point of the protein sample) corresponds to the lowest point. Two grooves or lowest points can be seen where the lowest point to the left did not change depending on the addition of the ligand, whereas the second lowest point showed ligand-dependent change in Tm during temperature increase. From the analysis of the second lowest point, stabilization of 2–3 degrees was obtained when the ligands were added to the protein (black line) as compared to the protein with ligand (green line, control sample). All measurements were setup in duplicates. The effect of Compound BCL3ANT on the Tm of GST-Bcl-3-ARD was also measured at an 0.1 mg/mL GST-Bcl-3-ARD in 10 mM HEPES, pH 7.4 with 1.9 mM Compound XY. The measurement was done once in duplicate.

### Western blot analysis

Cells were placed on ice and washed once with cold PBS before being harvested in cold 1 × lysis buffer (50 mM Tris–HCl pH 7.4, 150 mM NaCl, 1% Triton-x-100, complemented with 40 μl/ml complete protease inhibitors (Roche Applied Science). Lysates were cleared by centrifugation at 12,000 × g for 10 min at 4 °C and protein content was determined using a Bradford assay according to manufacturer’s instructions (Thermo Fisher Scientific). Equal amounts of protein were electrophoretically separated on 10% SDS/polyacrylamide gels and proteins were transferred onto Immobilion-FL polyvinylidene difluoride (PVDF) membranes (Millipore). Membranes were blocked with 5% non-fat milk in PBS-T for 1 h at room temperature followed by overnight incubation at 4 °C with primary antibodies against α-tubulin (1:4000, Sigma), Bcl-3 (1:500, Santa Cruz), cyclin D1 (1:1000, Abcam). Primary antibodies were detected with horseradish peroxidase-labeled secondary antibodies (1:5000, Dako). Chemiluminescence was detected with a charge-coupled device camera (Fujifilm).

### Immunofluorescent staining

Cells cultured on cover slips were washed twice with PBS and fixed for 4 min using 4% paraformaldehyde in PBS. Following fixation, cells were permeabilized using 0.25% Triton-x-100 in PBS for 10 min. After permeabilization, cells were washed three times in PBS and blocked for one hour in 1% bovine serum albumin (BSA) in PBS. Thereafter, cells were incubated for one hour with primary antibody in PBS followed by washing with PBS and incubation with Alexa Fluor 546 conjugated antibodies (Molecular probes) in PBS. Cover slips were mounted on object slides using Vectashield with diamidino-2-phenylindole (DAPI) (Vector Laboratories). Images were captured using a 40X oil objective on the Zeiss LSM 710 Confocal System (Zeiss).

### Real-time quantitative PCR

Cultured cells were washed in cold PBS and total RNA was extracted using the RNeasy extraction kit (5 Prime) according to manufacturer’s instruction. The purity of RNA was analyzed and quantified using a Nanodrop spectrophotometer (Saveen Werner) and cDNA synthesis was performed using qPCR cDNA synthesis kit (Stratagene) following manufacturer’s instructions. RT-qPCR runs were performed in the Mx3005P real-time thermocycler (Stratagene) with the following program: 2 min 50 °C, 10 min at 95 °C, followed by 40 three-step cycles consisting of 95 °C for 20 s, 60 °C for 30 s, and 72 °C for 1 min. The following primers were used:

- Bcl-3 sense 5’-TATTGCTGTGGTGCAGGGTA-3’
- Bcl-3 anti-sense 5’-CACCACATTACCGTCTGTGG-3’
- Cyclin D1 sense 5’-GGCGGAGGAGAACAAACAGA-3’
- Cyclin D1 anti-sense 5’-TGGCACAAGAGGCAACGA-3’
- GAPDH sense 5’-TGCACCACCAACTGCTTAGC-3’
- GAPDH anti-sense 5’-GGCATGGACTGTGGTCATGAG-3’

### Cell proliferation assay

Cell proliferation was measured using the WST-1 assay (Roche). In brief, equal number of cells were seeded on 96-well flat-bottom plates in 100 μl of complete cell culture medium for 24 h prior to drug treatments. After 24–96 h of drug treatment, 10 μl of WST-1 reagent was added to each well and plates were incubated for 3 h at 37 °C. Absorbance was measured at 450 nm using the Synergy 2 HT Multi-Mode Microplate Reader (Biotek).

### DNA fragmentation assay

Mel Ju and SK-MEL-3 cells were seeded in 6-well plates at a density of 2 × 10^5^ cells per well and incubated overnight. Medium was changed to fresh medium supplemented with or without compounds. After 48 h the medium was collected and centrifuged at 400 × g for 5 min. Meanwhile, adherent cells were washed with PBS, trypsinized, suspended in complete medium and centrifuged at 400 × g for 5 min. After aspirating medium, the cell pellets from adherent and suspended cells were washed in PBS and centrifuged at 400 × g for 5 min. Cell pellets were carefully resuspended in 150 μl PBS before 1.35 ml 70% EtOH was added to fix the cells at 4 °C overnight. The following day, cells were centrifuged, resuspended in PBS, and centrifuged at 400 × g for 5 min. Cell pellets were resuspended in 50 μl PBS supplemented with 1 μg/ml DAPI (Sigma Aldrich) and 0.1% Triton-x-100. Cells were analysed using the DNA fragmentation protocol on the NucleoCounter 3000 (Chemometec).

### Half-maximal inhibitory concentration (IC_50_) determination

The half-maximal inhibitory concentration (IC_50_) was determined by seeding equal number of cells on 96-well plates. Following attachment, medium was replaced with fresh medium with or without BCL3ANT at ascending concentrations and cells were incubated for 5 days. After 5 days the WST-1 assay was performed and cells were incubated for 3 h at 37 °C with WST-1 reagent before absorbance was measured at 450 nm using the Synergy 2 HT Multi-Mode Microplate Reader (Biotek). Data is presented as IC50 (50% reduction in cell viability).

### Viability assay

Mel Ju and SK-MEL-3 cells were seeded in 6-well plates at a density of 5 × 10^4^ cells per well and incubated for 24 h. At indicated time points cells were washed with PBS, trypsinized for 5 min and resuspended in complete medium before being centrifuged for 5 min at 400 × g. Cell pellets were resuspended in PBS, centrifuged, and counted using the “Viability and Cell Count” protocol on the NucleoCounter NC-3000 (Chemometec).

### Cell cycle analysis

Cells were seeded on 6-well plates at a density of 2 × 10^5^ cells per well and incubated overnight. Medium was changed to fresh medium supplemented with or without A27. After 48 h medium was collected and centrifuged at 400 × g for 5 min. Adherent cells were washed with PBS, trypsinized, and suspended in complete medium before being centrifuged for 5 min at 400 × g. After aspirating medium from the cell pellet, cells were washed in PBS and centrifuged for 5 min at 400 × g. Cell pellets were carefully resuspended in PBS before 70% ethanol was added and cells were fixed overnight at 4 °C. Fixed cells were centrifuged for 5 min at 400 × g and resuspended in PBS supplemented with 1 μg/ml DAPI (Sigma Aldrich) and 0.1% Triton-x-100. Cells were analysed using the “fixed cell cycle-DAPI” protocol on the NucleoCounter NC-3000 (Chemometec).

### Animal models

NUDE mice were purchased from Janvier Laboratory (France). The protocol for the in vivo metastasis assay on mice has been approved by the Center of ethical committee on animal experiments in Sweden "Centrala försöksdjurnämnden". Ethical Dnr: M129-15. All animals were maintained under specific pathogen-free conditions at the Medicon Village animal facility at Lund University. All animal experiments were performed according to the national and international guidelines of the European Union. All mice were housed under pathogen-free conditions in the animal facility and received autoclaved water and food. Ten control and ten BCL3ANT treated 6-week-old mice were used in this study. The mice were subcutaneously transplanted with 1.10^6^ cells/ml of SK Mel 3 melanoma cell lines. Six days after transplantation, the mice were treated intraperitoneally (IP) with 10 mg/kg BCL3ANT or vehicle control. The vehicle solution used to dissolve BCL3ANT and as a control was composed by 2% DMSO in PBS. The mice were treated every 3 days with BCL3ANT or vehicle and sacrificed after 16 days post transplantation. Tumor volume was measured using CALIPER. Treatment was repeated every third day until day 16. The mice were euthanized by exposure to CO_2_ without removing animals from their home cage.

### Statistical and survival analysis

Expression levels of Bcl-3 and survival data were gathered for melanoma patients with metastases, excluding those with brain metastases, from three pivotal studies [31–33]. Z scores for Bcl-3 expression were calculated for each dataset. Bcl-3 expression was categorized as low for negative z-scores and high for positive z scores. The data were visualized using GraphPad Prism 9. Data represented are mean ± SEM of at least three separate experiments. Statistical comparisons were carried out by student´s T-test and significance is indicated by *p* > 0.05, *p ≤ 0.05, **p ≤ 0.01, and ***p ≤ 0.001.

## Results

### Screening for small molecules that interfere with Bcl-3-mediated cyclin D1 activity

**Fig. 1:**
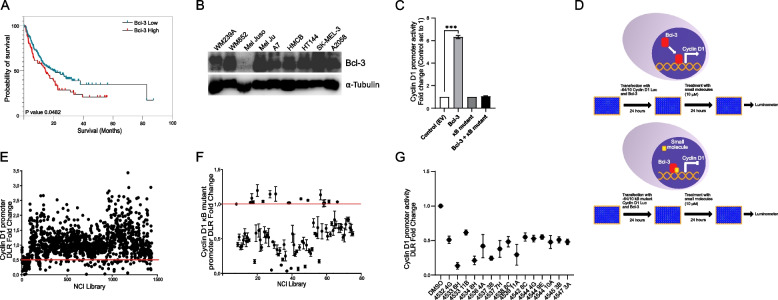
Screening for small molecules that interfere with Bcl-3-mediated cyclin D1 activity. **A** Survival curve of melanoma patients with metastasis stratified by Bcl-3 expression level. Patients are categorized into two groups: Bcl-3 high (positive z scores) and Bcl-3 low (negative z scores), based on the calculated z-scores for Bcl-3 expression from three pivotal studies. The x-axis represents the survival time in months, while the y-axis depicts the proportion of patients surviving. The survival differences between the two groups were evaluated, providing insights into the impact of Bcl-3 expression levels on the survival of melanoma patients with metastases. **B** Lysates prepared from human melanoma cell lines were subjected to Western blot analysis of Bcl-3 and α-Tubulin. **C** The effects of Bcl-3 on cyclin D1 promoter activity. Mel Juso cells were cotransfected with empty vector (EV), Bcl-3, cyclin D1 κB mutant, together with reporter plasmid cyclin D1 luciferase and Renilla-luciferase. Cyclin D1 promoter activity was measured using the Dual Reporter Luciferase assay. **D** Schematic overview of the cell-based luciferase reporter assay as a high throughput screening model. This model utilizes a reporter construct containing only the part of the cyclin D1 promoter (-64/ + 10) that can be occupied by Bcl-3, or a cyclin D1 κB mutant as a control. The upper panel shows the Bcl-3-specific κB site on the cyclin D1 promoter. The lower panel shows the ligands that were selected that could inhibit Bcl-3-mediated cyclin D1 promoter activation. **E** Small molecule screening using 1368 small molecules from a chemical library from the National Cancer Institute. Mel Juso cells were transfected for 24 h with Bcl-3 and cyclin D1, followed by treatment with 10 μM of each small molecule. After 24 h of treatment, samples were analyzed using a DLR luciferase assay. Ligands were selected based on the selection criterion of a 50% or greater reduction in Bcl-3-mediated cyclin D1 promoter activity. **F** Mel Juso cells were transfected with Bcl-3 and cyclin D1 κB mutant constructs and subjected to treatment with the 79 selected ligands from the first screen. The selection criterion for screen 2 was that no reduction in cyclin D1 promoter activity could be observed in the presence of the cyclin D1 κB mutant. **G** Mel Juso cells were transfected with Bcl-3 and cyclin D1 (-64/ + 10) constructs and treated with 15 ligands (selected after screen 1 and screen 2). Bcl-3-mediated promoter activity in the presence of each ligand was analyzed using the DLR luciferase assay. All data are presented as the mean ± SEM from three independent experiments. Statistical significance was assessed using Student’s t test

Initially, we investigated the survival curve of melanoma patients with metastasis stratified by the Bcl-3 expression level and found that the reduced survival rate of melanoma patients with metastases correlated with high levels of Bcl-3 expression (Fig. 1A). Analysis of Bcl-3 expression levels in nine human melanoma cell lines showed high levels of Bcl-3 expression in all except Mel Juso cells (Fig. 1B). We decided to use high-throughput screening of small molecules for the identification of Bcl-3-mediated cyclin D1 expression. To avoid interfering with the endogenous high expression of Bcl-3, we selected Mel Juso cells to control the level of Bcl-3 overexpression in these cells. A cell-based luciferase reporter assay was performed as a high-throughput screening model by utilizing a reporter construct containing only the part of the cyclin D1 promoter (-64/ + 10) that can be occupied by Bcl-3. As a control, a mutant form of the Bcl-3 binding site at the cyclin D1 promoter (-64/ + 10-mut) was selected for this screening assay. The procedure of the assay is presented in Fig. 1D. As expected, activation of cyclin D1 could be observed with -64/ + 10 cyclin D1 but not with the -64/ + 10-mut cyclin D1 κB mutant plasmid (Fig. 1C). Screening 1368 small molecules from a chemical library of the National Cancer Institute resulted in the identification of 79 small molecules that could decrease cyclin D1 promoter activity by 50% (Fig. 1E). After repeating the screening process with the 79 identified small molecules (Fig. 1F), we found 15 small molecules that could effectively reduce cyclin D1 promoter activity (Fig. 1G).

### Identification of the Bcl-3 antagonist that inhibits cyclin D1 expression

**Fig. 2:**
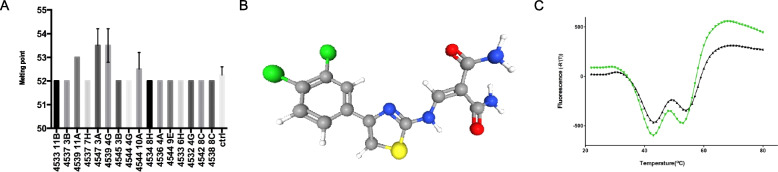
Identification of the Bcl-3 antagonist that inhibits cyclin D1 expression. **A** The binding of 4547 3A (BCL3ANT) and 4539 4G to the Bcl-3 ARD causes a significant shift in melting temperature compared to the other compounds. Fluorescence based screening of ligands toward GST-Bcl-3-ARD, where ARD stands for ankyrin repeat domain. Analysis of the melting point for the ligands where the binding of 4547 3A (BCL3ANT) to the protein sample caused the largest shift in melting temperature of 2–3 degrees. **B** Chemical structure of BCL3ANT consisting of 2-(((4-(3,4-dichlorophenyl)-1,3-thiazol-2-yl) amino) methylene). **C** The green line represents the control consisting of Bcl-3 ARD protein without the ligand, and the red line represents Bcl-3 ARD in the presence of ligand (BCL3ANT) causing a shift in melting temperature from 51 °C to 54 °C

To reduce the number of small molecules, a fluorescence-based thermal shift assay was performed to analyze the linkage between the Bcl-3 protein and ligand binding. For this experiment, the ankyrin repeats of the Bcl-3 protein, which is the part of the protein that can interact with p50 and p52, were produced and purified. The fluorescence-based thermal shift assay identified 2 ligands among 15 to achieve a temperature shift up to 3 °C compared to the control (Fig. 2A). These two ligands were 2-(((4-(3,4-dichlorophenyl)-1,3-thiazol-2-yl) amino) methylene) malonamide hydrochloride and methoxyellipticine. In silico analysis using the GLIDE program revealed that 2-(((4-(3,4-dichlorophenyl)-1,3-thiazol-2-yl) amino) methylene) showed a better docking score than methoxyellipticine. The 2-(((4-(3,4-dichlorophenyl)-1,3-thiazol-2-yl) amino) methylene) compound is synonymous with NSC-659509. The molecular formulation of NSC-659509 is C_13_H_11_C_l3_N_4_O_2_S (Fig. 2B) with a molecular weight of 394 Da. We also assessed the stability of the Bcl-3 protein in the presence of NSC-659509 with a thermal stability assay. At 1.9 mM, NSC-659509 showed a ΔT_m_ of 4.1 °C, a sample with Bcl-3 without ligand was used as the reference control. To confirm these results, the unfolding of the Bcl-3 protein indicated by the increase in the fluorescence of the fluorophore SYPRO Orange was investigated. As is shown in Fig. 2C, stabilization of 3 degrees was obtained when the ligands were added to the protein (black line); protein without ligand was used as the control (green line, Fig. 2C).

**Fig. 3:**
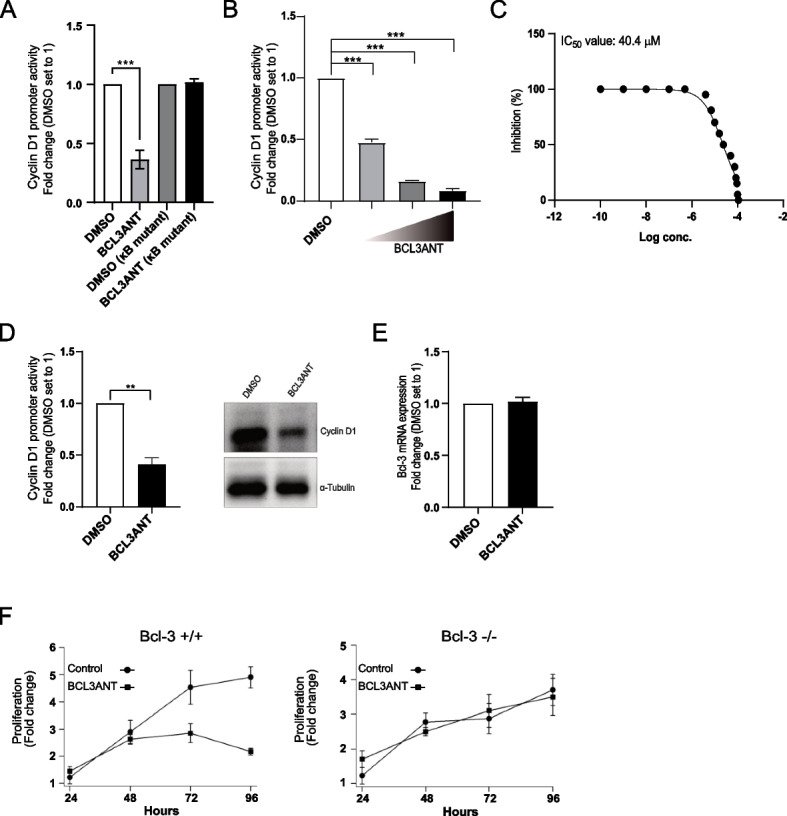
Characterization of the Bcl-3 antagonist that inhibits cyclin D1 expression. **A** Mel Ju cells were transfected with cyclin D1 or cyclin D1 κB mutant promoter together with Renilla Luciferase. Following transfection, cells were treated with 50 μM BCL3ANT for 24 h and analyzed for Bcl-3-mediated cyclin D1 promoter activity using the luciferase assay. **B** Mel Juso cells were cotransfected for 24 h with the Bcl-3 and cyclin D1 promoters, followed by 2a 4-h treatment with 10 μM, 20 μM, or 30 μM BCL3ANT, or DMSO as a control. Following incubation with treatment, Bcl-3-mediated cyclin D1 promoter activity was analyzed by luciferase assay. **C** The half-maximal inhibitory concentration (IC_50_) of Mel JU was determined after measuring cell viability using a WST-1 assay. **D** (Left) mRNA from Mel Ju cells was prepared after 24 h of treatment with or without 50 μM BCL3ANT and subjected to real-time quantitative PCR (qPCR) analysis of cyclin D1 and the housekeeping gene GAPDH. (Right) Lysates prepared from Mel Ju cells following 24-h treatment with or without 50 μM BCL3ANT were subjected to Western blot analysis of cyclin D1 and α-tubulin. **E** mRNA from Mel Ju cells was prepared after 24 h of treatment with or without 50 μM BCL3ANT and subjected to real-time quantitative PCR (qPCR) analysis of Bcl-3 and the housekeeping gene GAPDH. **F **SiRNA-induced knockdown of Bcl-3 or siRNA-control knockdown cells in immortalized keratinocytes were treated with DMSO or 50 μM BCL3ANT for 24-96 h before being subjected to a WST-1 assay to measure cell proliferation. Data are presented as the mean ± SEM from three independent experiments. Statistical significance was assessed using Student’s t test

We confirmed that NSC-659509, hereafter referred to as BCL3ANT, could interfere with Bcl-3-mediated cyclin D1 promoter activity, but this effect was not observed when the cyclin D1 κB mutant was expressed (Fig. 3A). Additionally, BCL3ANT reduced cyclin D1 luciferase activity in Bcl-3-overexpressing melanoma cells in a concentration-dependent manner (Fig. 3B). We treated Mel Ju cells with increasing concentrations of BCL3ANT and found that the IC_50_ value was 40.4 μM (Fig. 3C). Furthermore, we showed that Mel Ju cells treated with BCL3ANT had reduced cyclin D1 mRNA and protein levels compared to control-treated cells (Fig. 3D). No differences could be observed in Bcl-3 mRNA levels after treatment of Mel Ju cells with BCL3ANT compared to DMSO-treated cells (Fig. 3E). SiRNA-induced knockdown of Bcl-3 in immortalized keratinocytes [24] and treatment with BCL3ANT did not affect cell growth and cell proliferation of the cells (Fig. 3F right panel) compared to siRNA-control cells (Fig. 3F left panel). In conclusion, these results suggest that BCL3ANT-mediated cyclin D1 repression occurs at the promoter level.

### BCL3ANT inhibits melanoma cell proliferation and migration

**Fig. 4:**
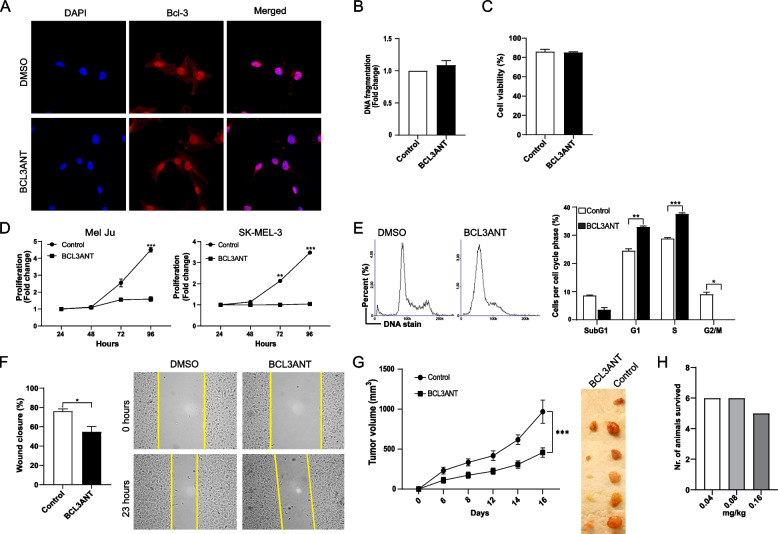
BCL3ANT inhibits melanoma cell proliferation and migration. **A** Mel Ju cells were treated with DMSO or 50 μM BCL3ANT for 24 h before being stained with antibodies against Bcl-3 (red) and 406-diamidino-2-phenyl indole DAPI (blue) and imaged using confocal microscopy. **B** Mel Ju cells were treated with DMSO or 50 μM BCL3ANT for 24 h and the percentage of fragmented DNA and (C) cell viability were analyzed using a NucleoCounter. **D** Mel Ju and SK-MEL3 cells were treated with DMSO or 50 μM BCL3ANT for 24–96 h before being subjected to a WST-1 assay to measure cell proliferation. **E** Mel Ju cells were treated with DMSO or 50 μM BCL3ANT for 48 h and the cell cycle was assessed using a NucleoCounter. Representative histograms showing the cell cycle phases from Mel Ju cells after treatment with DMSO or BCL3ANT. **F** Mel Ju cells were seeded onto a 6-well plate and incubated for 24 h to adhere to the plate. A wound was created using a 1 ml pipet tip before the cells were treated for 23 h with DMSO or 50 μM BCL3ANT. An image of the wound was taken at 0 h and 23 h post treatment, and the area of the wound was calculated to determine cell migration. **G** NUDE mice were transplanted with SK-MEL3 melanoma cells and 6 days post-transplantation, mice were treated with 10 mg/kg BCL3ANT or vehicle control every 3 days until Day 16 when they were sacrificed. Tumor volume was measured using a CALIPER. **H** Six mice per group were treated with 0.04, 0.08, or 0.16 mg/kg BCL3ANT once and observed for 30 days for the development of adverse effects. All data are presented as the mean ± SEM from three independent experiments. Statistical significance was assessed using Student’s t test

After establishing that BCL3ANT interferes with the expression of cyclin D1, we wanted to examine the effect of Bcl-3 inhibition in melanoma cells. Since Bcl-3 is predominantly a nuclear protein but can be found within the cytoplasm of cells, we initially analyzed whether inhibition of Bcl-3 affects its cellular localization. Treatment of Mel Ju cells with BCL3ANT did not alter the subcellular localization of Bcl-3 compared to control treated cells (Fig. 4A). Additionally, no differences were found in the amount of DNA fragmentation or cell viability after treatment of Mel Ju cells with BCL3ANT within 24 h of treatment (Fig. 4B and C), indicating that cell survival is not affected by BCL3ANT treatment. Next, cell proliferation was monitored in the presence of BCL3ANT. We treated Mel Ju and SK-MEL-3 cells with BCL3ANT for 72 h, and observed a significant reduction in the cell proliferation rate compared to that of control cells (Fig. 4D). Accordingly, we observed an accumulation of cells in the G1 and S phase of the cell cycle after treatment of cells with BCL3ANT in comparison to control cells (Fig. 4E). To examine whether Bcl-3 inhibition affected cell migration, a wound healing assay was performed. Melanoma cells treated with BCL3ANT migrated more slowly than control-treated cells (Fig. 4F). Finally, we transplanted melanoma cells into immunocompromised nude mice and treated these animals with control or BCL3ANT for 16 days. A significant delay in tumor growth was observed in animals treated with BCL3ANT compared to control-treated animals (Fig. 4G). According a database [dtp.cancer.gov/databases], the animals were treated with 0.04 mg/kg, 0.08 mg/kg, or 0.16 mg/kg of BCL3ANT, and all animals beside one from 0.16 mg/kg were survived at each of the dose levels (see https://dtp.cancer.gov/databases_tools/data.search.htm) (Fig. 4H). Taken together, these data suggest that treatment of melanoma cells with BCL3ANT reduces cell proliferation by blocking the G1-to-S cell cycle transition via inhibition of cyclin D1 expression and limits the growth of the cells in vivo.

## Discussion

The effectiveness of systemic therapies for metastasized malignancies is still very limited. There is an urgent need for the development of drugs, without any major side effects that can cure patients or prolong their survival. Malignant melanoma is responsible for 80% of skin cancer-related deaths. In Europe, almost 100,000 individuals are diagnosed each year. The annual increase in incidence rate varies between populations, but in general, a doubling of melanoma incidence every 10–20 years has been estimated https://www.globaldata.com/store/report/melanoma-market-analysis/. Metastatic melanoma is usually an incurable condition that, prior to the introduction of immunotherapy, had a median survival rate of 6–9 months. However, since the approval of therapeutic cytokines and immune checkpoint inhibitors, the survival of metastatic melanoma patients has significantly improved, with a median survival rate of approximately 6 years for those treated with a combination of immunotherapy agents [34, 35]. Nevertheless, immune checkpoint inhibitor resistance is commonly seen in melanoma patients, with almost 40% acquiring resistance to immunotherapy within 3 years [36, 37]. Recurrence of melanoma can occur locally or at distal sites. Local recurrence, which often occurs months to years after surgical removal, often indicates that the tumor has metastasized, and these patients have a low rate of survival. Most cases of recurrences (55% to 79%) are discovered with 2 years, whereas 65% to 85% are discovered within 3 years after the initial diagnosis of the primary tumor (https://www.curemelanoma.org/about-melanoma/melanoma-staging/melanoma-survival-rates).

Earlier studies including research from our laboratory have shown that the growth and metastasis of melanoma cells are regulated by elevated expression of oncogene B-cell CLL/lymphoma 3 (Bcl-3) [19, 25, 29]. Bcl-3 was originally identified as a t(14;19) chromosomal translocation in a subgroup of B-cell chronic lymphocytic leukemia [14]. High levels of Bcl-3 expression and activation have been detected in different types of human cancer, such as skin cancer, leukemia, hepatocellular carcinoma, breast cancer, colon cancer, ovarian cancer and prostate cancer [38]. Increased levels of Bcl-3 in these cancer forms correlated with poor prognoses of patients. In our previous human patient studies, we also proposed that Bcl-3 expression and localization can be used as a diagnostic marker in different types of cancer [20, 24]. Bcl-3 has also recently been shown to promote a cancer stem cell phenotype in tumor cells [21], resistance to chemotherapy [39], and facilitation of tumor cell migration and invasion [40–42]. Bcl-3 was also shown to induce the expression of the immune checkpoint PD-L1 expression in tumor cells [43]. The role of Bcl-3 in cancer hallmarks has been published recently for colorectal cancer [44]. Based on these discoveries, the overall aim of the present study was to discover, develop and characterize Bcl-3 inhibitors as anticancer drugs for human cancer patients.

In the current study, we focused on targeting Bcl-3-mediated cyclin D1 expression, which is a well-established mechanism. A high-throughput screening model was employed to test 1368 small molecules from a chemical library of the National Cancer Institute. This screening identified 79 small molecules, which were further narrowed down to one compound, BCL3ANT, through additional experiments. BCL3ANT was selected for further studies, as it demonstrated efficacy in reducing cyclin D1 promoter activation, cyclin D1 mRNA levels, and protein levels compared to those in cells treated with a control substance. This result indicates that BCL3ANT-mediated cyclin D1 repression occurs at the promoter level. Since BCL3ANT interfered with the activity of cyclin D1, we examined melanoma cell proliferation by using two different cell lines. A significant reduction in cell proliferation could be observed in both cell lines compared to control cells. Cell cycle analysis of these cells demonstrated an accumulation of cells in the G1 and S phases of the cell cycle after treatment with BCL3ANT. Previously, Bcl-3 expression was shown to affect the cell migration and invasion of melanoma cells. In the present study, compared to the control, BCL3ANT reduced the migration capacity of cancer cells. In addition, in mice transplanted with melanoma cells, treatment with BCL3ANT significantly reduced the growth of tumor cells compared to that in control group.

Previous studies searching for inhibitors in the atypical NFκB signaling pathways identified a specific inhibitor of androgen receptor and p52 interaction resulting in reduced levels of cyclin D1 expression used for the treatment of castration-resistant prostate cancer [45]. A small-molecule inhibitor against pirin inhibited the interaction between pirin and Bcl-3 in melanoma, leading to inhibition of melanoma cell migration. This mechanism was mediated via downregulation of snail protein and blockade of EMT transition [46]. The benefits of using Bcl-3 blocking instead of recently approved CDK4/6 inhibitors are that targeting Bcl-3 will only affect metastatic melanoma cells, while CDK4/6 inhibitors affect all actively dividing cells. Furthermore, the use of CDK4/6 inhibitors has been shown to cause hematological and gastrointestinal adverse events [47, 48]. Additionally, therapy resistance remains a hurdle in treating metastatic melanoma patients, and even though the CDK4/6 pathway is often dysregulated in melanoma patients, clinical data have suggested an intrinsic resistance of melanoma to CDK4/6 inhibitors [49–51]. In the present study, we focused on melanoma as a proof of concept and identified a lead molecule that could interfere with Bcl-3-mediated cyclin D1 expression as well as proliferation and migration in melanoma cells. Future studies need to focus on constructing analogs of BCL3ANT for lead optimization and preclinical verification studies, such as PK/PD and ADME studies. In such experiments, the key issues of the tissue distribution of selected compounds also needs to be addressed.

### Supplementary Information

**Additional file 1: Supplementary Figure 1.**

## Data Availability

The datasets used and/or analysed during the current study are available from the corresponding author on reasonable request.
