# Supplementary material for: Discovery of a small molecule that inhibits Bcl-3-mediated cyclin D1 expression in melanoma cells
Source: BMC Cancer. 2024 Jan 18;24:103. doi: 10.1186/s12885-023-11663-y (PMC10795364; doi:10.1186/s12885-023-11663-y)

Supplementary Figure 1

Original blots: Fig. 1B

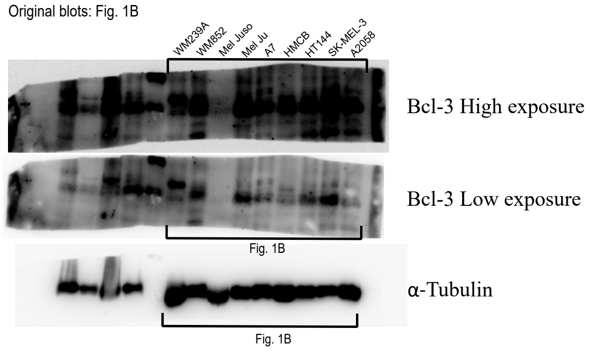

Original blots: Fig. 3D

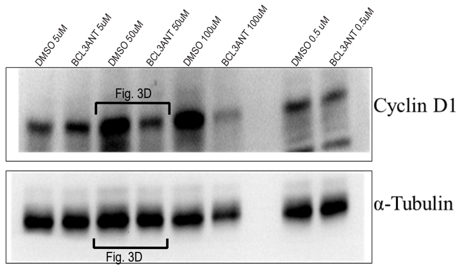

Supplement: Supplementary file 1 — Additional file 1: Supplementary Figure 1. [file 12885_2023_11663_MOESM1_ESM.pdf]
